# The Mediating Role of Sleep Quality in the Association between Urinary Incontinence and Depressive Symptoms

**DOI:** 10.1093/geroni/igaf122.3105

**Published:** 2025-12-31

**Authors:** Seo-Yun Choi, Yuri Jang, Ju Hyun Kim

**Affiliations:** University of Southern California, Los Angeles, California, United States; University of Southern California, Los Angeles, California, United States; Chungnam National University, Daejeon, Republic of Korea

## Abstract

Urinary incontinence (UI) is a prevalent health issue in the older adult population. Although the negative mental health impact of UI is widely known, the underlying mechanisms remain unclear. Given that UI’s physical and emotional discomfort make older adults prone to impaired sleep, we conceptualized sleep quality as a mediator between UI and depressive symptoms. The target group of the present study is older Korean Americans living in subsidized senior housing, who are part of an older immigrant population with socioeconomic disadvantages and health disparities. Using survey data collected from older Korean-American residents of subsidized senior housing in Los Angeles in 2023 (N = 306, Mean age = 79.4, SD = 6.65), we examined the direct effect of UI on depressive symptoms and the mediating role of sleep quality. UI was indicated by the frequency of experiencing involuntary urine leakage (never/infrequent/frequent), sleep quality was self-rated on a scale from excellent to poor, and depressive symptoms were assessed using the Patient Health Questionnaire (PHQ-9). Multivariate analyses showed the significant direct impact of UI on depressive symptoms. The Hayes PROCESS macro provided supportive evidence for the indirect effect of UI on depressive symptoms through sleep quality (B [SE] = .53 [.15], bias-corrected 95% Confidence Interval = [.24, .85]). The indirect effect accounted for 26.9% of the total effect. Sleep quality was identified as an important pathway through which UI undermines mental health. The study’s findings highlight the importance of incorporating sleep-focused strategies into mental health interventions for those affected by UI.

